# Sustainable solutions to barriers of point-of-care diagnostic testing services in health facilities without laboratories in the Bono Region, Ghana: a qualitative study

**DOI:** 10.1186/s12875-024-02406-4

**Published:** 2024-05-22

**Authors:** Monica Ansu-Mensah, Vitalis Bawontuo, Desmond Kuupiel, Themba G. Ginindza

**Affiliations:** 1 Discipline of Public Health Medicine, School of Nursing and Public Health, University of KwaZulu-Natal, Durban, 4001 South Africa; 2 The University Clinic, Sunyani Technical University, Sunyani, Ghana; 3 Department of Health Services Management and Administration, School of Business, SD Dombo University of Business and Integrated Development Studies, Wa, Ghana; 4 Faculty of Health Sciences, Durban University of Technology, Durban, 4001 South Africa; 5 Cancer & Infectious Diseases Epidemiology Research Unit (CIDERU), College of Health Sciences, University of KwaZulu-Natal, Durban, South Africa

**Keywords:** Barriers, Potential solutions, POC diagnostic testing, Implementation, Bono Region

## Abstract

**Background:**

A sustainable point-of-care (POC) diagnostic testing implementation in low-resourced facilities enhances quick diagnostic investigation and halts unnecessary referrals. This study identified the barriers impeding the implementation of POC diagnostic testing in health facilities without laboratories in the Bono Region of Ghana; as well as explored potential solutions that could enhance the accessibility and effectiveness of POC diagnostic testing, ultimately improving the quality of healthcare delivery.

**Methods:**

A total of twenty-eight participants were purposively selected from health facilities in low-resourced settings in the Bono Region for a descriptive qualitative study. Of the twenty-eight participants, seventeen including ten healthcare providers from CHPS facilities, six district health depot managers, and one regional depot manager were engaged in in-depth interviews. Additional eleven including nine healthcare providers and two district depot managers were also engaged in focus group discussions. NVivo version 12 software was employed for condensation, labelling, and grouping of themes. Data was analysed narratively.

**Results:**

Work overloads, limited POC testing services, stock-outs of POC tests at the facilities, and supply-related challenges of POC test kits were identified as major barriers to POC testing services. To solve these barriers, adequate funding, an effective delivery system, stakeholders’ engagement and advocacy, and in-service and refresher training courses were suggested as potential solutions to POC diagnostic testing services implementation by the stakeholders.

**Conclusions:**

This study’s findings emphasize the need to address the barriers hindering the implementation of POC diagnostic testing in health facilities without laboratories in the Bono Region of Ghana. The suggested solutions provide a roadmap for improving the accessibility and effectiveness of POC testing, which has the potential to enhance the quality of healthcare delivery, reduce unnecessary referrals, and ultimately improve patient health outcomes in underserved settings.

**Supplementary Information:**

The online version contains supplementary material available at 10.1186/s12875-024-02406-4.

## Introduction

The significance of timely and accurate diagnostic testing cannot be overstated in the quest to provide effective healthcare services, particularly in resource-constrained settings. Point-of-care (POC) diagnostic testing promises to revolutionize healthcare delivery, particularly in regions where access to fully equipped laboratories is limited [1–6]. Ghana, like many other low-andmiddle-income countries, faces substantial challenges in providing accessible and efficient diagnostic services, particularly at the primary healthcare (PHC) level, often resulting in delayed diagnosis, mismanagement of diseases, and unnecessary referrals to higher-level healthcare facilities [7–11].

Healthcare facilities without laboratories in Ghana, such as Community-Based Health Planning and Services (CHPS) centers, play a pivotal role in the healthcare ecosystem [12]. However, the lack of laboratory infrastructure in these settings has posed significant obstacles to delivering quality healthcare. These facilities often rely on external laboratories for diagnostic services, causing delays in patient care and increasing the burden on already overwhelmed healthcare providers. To address the diagnosis challenges faced by PHC facilities such as CHPS facilities in Ghana, the World Health Organisation (WHO) through its essential in-vitro diagnostics list (EDL) recommended diagnostic tests (e.g. glucose, haemoglobin (Hb), hepatitis B surface antigen (Hep B), blood typing, and HIV1/2 antibodies) that can be used in health facilities without laboratories for adoption by countries [13].

These POC diagnostics adhere to WHO quality-ASSURED (affordable, sensitive, specific, user-friendly, rapid and robust, equipment-free, and deliverable) recommendations, and are essential for disease diagnosis and increasing access to healthcare in resource-limited settings [14, 15]. Moreover, these POC diagnostics are essential and are one of the several vehicles of attaining the Universal Health Coverage (UHC) by 2030 [13, 16–18]. Despite this essential role of POC diagnostics in healthcare access, their effective implementation often faces barriers [19]. These barriers are multifaceted and may be similar or country-specific depending on each country’s income status [4, 20, 21]. While some countries are challenged with cultural, financial, or religious barriers, others are faced with economic, political, or health system barriers [4, 22–26]. Unlike high-income countries, LMICs are mostly challenged with financial constraints which usually affect human resources, supply chain deficiencies, geographical accessibility, POC test shortages, and affordability of testing services [4, 24, 27–30].

In Ghana, the existing literature on POC diagnostics shows a significant research gap in the Bono Region. Although several studies have been conducted on POC diagnostics implementations in Ghana, these investigations mostly involved Northern Ghana with a few in Southern Ghana. Whiles Kuupiel et al.; Ward et al.; Chatio et al. and Sinha et al. investigated various aspects including POC diagnostic accessibility, availability, price, supply chain management, sensitivity, and specificity in Northern Ghana (Upper East and Northern Region) [31–36], Osei et al. and Dassah et al. investigated diagnostic accuracy, factors associated with failure to screen in Southern Ghana (specifically, Ashanti Region) [37, 38]. However, our previous study that involved the Bono Region revealed poor availability and low stock level of POC diagnostic tests [5]. Recognising this research gap and the need to address the availability and accessibility of POC diagnostic services, we embarked on a comprehensive study aimed at identifying the barriers impeding the implementation of POC diagnostic testing in health facilities without laboratories within the Bono Region of Ghana. Furthermore, we sought to explore potential solutions that could enhance the accessibility and effectiveness of POC diagnostic testing, ultimately improving the quality of healthcare delivery.

## Materials and methods

### Study design and setting

A qualitative study was employed following a quantitative study to explore the availability, stock levels, and usage of POC tests at the CHPS facilities in the rural areas of the Bono Region [5]. The region is one of the newly created regions in Ghana with few hospitals. This region has been well described in the previous quantitative study. In that study, poor supply chain management compliance and low availability of POC tests were revealed in the region [5]. A cross-sectional study design using in-depth interviews and focus group discussion (FGD) was implemented to describe potential solutions to POC diagnostic testing barriers to support quality service delivery in low-resourced settings in the Bono Region of Ghana.

### Study participants and sampling

Study participants included in-charges of CHPS compounds, and district, and regional health depot managers. Nineteen [19] CHPS compound in-charges were purposively selected because of their roles in POC diagnostic services at the facility level. Additionally, eight [8] district health depot managers were conveniently selected from 12 participating health district depots in the region, based on willingness and availability to participate in the study. Finally, the regional health depot manager was purposively selected because of the supervisory role over district health depots, and manager for supplies of POC tests in the region. Thus, the study employed 28 participants and gathered sufficient data to explore barriers, and potential solutions to POC diagnostic testing to support quality service delivery in low-resourced settings in the Bono Region of Ghana.

### Data collection

In-depth interviews were conducted to collect data in July 2022 using 2 separate interview guides adapted from the Mashamba-Thompson et al. study [39]: one for 10 in-charges of CHPS facilities, and another one for 6 districts and 1 regional depot manager. Interviews were conducted until saturation was reached and no new data were emerging. Regarding FGD, a FGD guide was developed [40] which 2 research assistants with the PI used to conduct the FGD and collected data from 11 participants (9 from CHPS facilities and 2 from district depots) in August 2022. Both interview and FGD guides were prepared with sufficient probes and prompts to gather data on the POC diagnostic service barriers and how these barriers could be averted to ensure sustained POC diagnostic services in the low-resourced settings of the Bono Region. Interview and FGD dates, venues, and times were communicated to all participants beforehand to ensure commitment. The FGD was held in a ventilated room in one of the participating CHPS facilities. All interviews and FGD were audio recorded and field notes were taken. The study received informed consent from all participants.

### Data analysis

Audio-recorded interviews and FGD were transcribed verbatim by the Principal Investigator. Transcribed data was cleansed and exported to NVivo version 12 software for condensation, labelling, and grouping of themes [8]. In the analysis, 3 coders, merged similar codes and nodes to enable correct and proper interpretations of all the data. An inductive content analysis approach was used to code different patterns and review them to ensure coherency. The use of an open discussion strategy was employed to resolve coding disagreements. Emerging themes were then identified and continuously reviewed until a true reflection of the data set was seen. A member check was done to improve the accuracy, credibility, validity, and transferability of the study.

## Results

### Participants characteristics

Twenty-eight (28) people participated in the study included: 1 Regional Depot Manager, 8 District Depot Managers, and 19 Healthcare Professionals (HCPs). HCPs included in the study were 2 Physician Assistants, 4 Midwives, 1 Mental Health Officer, 3 General Nurses, 4 Health Assistants Clinical, and 5 Community Health Nurses (Table 1).

**Table 1:** Summary of study participants showing positions, and district

	Study site (district)	Positions of Participants	No. of Males	No. of Females	Total No. of Participants
**Depots**					
1.	Sunyani	Regional Depot Manager	1	0	1
2.	Banda, Berekum East, Dormaa East, Dormaa West, Jaman North, and Jaman South	District Depot Store Managers	6	2	8
**CHPS**					
3.	Berekum East, Dormaa Central, Dormaa West, Jaman North, Jaman South, Sunyani, Sunyani West, Wenchi, and Tain	Physician Assistants [2], Midwives [4] Mental Health Officers [1], General Nurses [3], Health Assistant Clinical [4], and Community Health Nurses [5].	6	13	19

The results are presented in line with the themes and sub-themes that emerged from the data analysis. These themes and sub-themes are presented in Table 2.

**Table 2:** Themes and categories

Themes	Categories
Barriers to POC diagnostic testing	• Work overload • Limited POC services • Stock-outs of POC test kits • Supply-related challenges of POC diagnostic test kits
Potential solutions to barriers to POC diagnostic testing implementation	• Adequate funding • Effective delivery system • Stakeholders’ engagement and advocacy • In-service and refresher training courses

### Barriers to POC diagnostic testing

The findings of this study revealed that implementing POC diagnostic testing in low-resourced settings of the Bono Region faces some challenges. The data analysis has grouped the barriers into the following sub-themes: work overload, limited POC services, stock-out of POC test kits, and supply-related challenges of POC diagnostic test kits.

#### Work overload

A detailed analysis of the data revealed that work overload of staff in low-resourced facilities affects the implementation of POC testing in rural settings. Participants reported that facilities were understaffed, thus few providers were left to perform multiple tasks. For instance, one participant indicated:

[…] *The facility is understaffed; the workload is difficult. We urgently need additional hands* […] (Interviewee 2).

Another participant explained:

[…] *I am the only person retrieving folders, doing testing, and serving drugs to patients. This is very worrisome. I am overloaded* […] (Interviewee 3).

Upon critical discussion, another reason for work overload was absenteeism among staff at the CHPS facilities. The analysis showed that frequent absenteeism caused work overload among a few staff who are at a post in the facilities. A FGD participant exclaimed:

[…] *Absenteeism is giving us hectic days at work. The work overload is unbearable!* […] (FGD Participant 2).

In response to frequent absenteeism, the analysis revealed that some staff live in the city (Sunyani) because there were no staff accommodations or decent houses for renting in rural communities. For this reason, fatigue and excess stress from travelling journeys were said to make it difficult for them to report to work every day. A FGD participant reported:

[…] *Uhm. There is no accommodation for staff and no decent houses for rent in this community* […] (FGD Participant 7).

Another FGD Participant added:

[…] *We live in the city, and reporting to work every day is very difficult due to increased stress, and fatigue from the journey* […] (FGD Participant 5).

Another reason the study found creating work overload in rural health facilities was lateness to work. It was reported that lateness was incomparable at CHPS facilities. Lateness affected the opening hours of the CHPS facilities and resulted in patients staying in long queues which caused work overload. A FGD participant indicated:

[…] *We come to work late and therefore do not start work on time. There is always a queue in the facility* […] (FGD Participant 1).

Another FGD participant interrupted:

[…] *Yes, opening hours are affected. We work for a few hours and close* […] (FGD Participant 5).

As to why staff reports to work late, participants expressed that because means of transport and the road networks were not good, coupled with insecurity on the road, staff arrived late to the facility and consequently, affected the opening times of the facility. A participant indicated:

[…] *The only means of transport in these communities is “Pragya” (tricycles). It works at specific times of the day because of the terrible road network and insecurity on the road* […] (FGD Participant 10).

A FGD participant indicated:

[…] *Drivers start working after 8 am and a long time is spent on the journey due to the deplorable nature of the road. We get to the facility late and close before 5 pm because we must catch the last transport back home* […] (FGD Participant 6).

Another FGD participant added:

[…] *Insecurity on the road is affecting travelling hours on these roads* […] (FGD participant 1).

#### Limited POC testing services

It was revealed in the analysis that some facilities were limited to some POC testing services due to various reasons. For instance, POC testing service may depend on proximity to a higher-level facility or staff strength to provide the service. It was indicated that a trained and qualified staff like a midwife is needed to handle maternal screening without which tests such as Hepatitis B, HIV, or syphilis will not be done in the CHPS facility. A FGD participant indicated:

[…] *we don’t attend to pregnant women and for that matter most of the maternal screening tests such as HIV/syphilis, Hep B, and Hb are not used here* […] (FGD Participant 1).

As explained by participants, POC testing was said to be restricted to only human chorionic gonadotropin (HCG) POC testing for antenatal screening because of a higher-level facility in proximity. A FGD participant submitted:

[…] *Our service is limited to Human Chorionic Gonadotropin because of the family planning services we offer* […] (FGD Participant 8).

A participant explained:

[…] *We are about a minute’s walk to a hospital. So, all other tests are run by the hospital to the extent that common malaria tests are done by the hospital. So POC implementation scale-up will be difficult* […] (Interviewee 4).

The study findings also indicated that lack of electricity supply contributed to inadequate staffing and subsequent limitation of POC testing service in low-resourced facilities. A participant indicated:

[…] *We have inadequate staff because there is no electricity in this community, so staff posted here reject the postings* […] (Interviewee 13).

Participants explained that because of electricity, midwives posted to rural-level facilities refused postings, and those who accepted postings requested transfers shortly. This phenomenon resulted in malaria testing only at some lower-level facilities. A participant indicated:

[…] *POC testing service here is limited to malaria because the facility is short of a midwife. We have staff accommodation for a midwife, but any midwife posted here goes back because of a lightning issue.* […] (Interviewee 2).

Again, in our analysis, new NHIA accreditation or renewal was another reason for limited POC testing services. It was mentioned that facilities without active NHIA accreditation have their POC test services limited to only programme commodities (malaria and HIV tests). A FGD participant commented:

[…] *We cannot render any POC testing service apart from malaria, and HIV because the facility is not accredited* […] (FGD Participant 7).

Another FGD participant reported:

[…] *We have undergone through NHIA renewal process for more than 3 months now and still have not received a renewal. We no longer use non-programme commodities* […] (FGD Participant 11).

Further probes into why CHPS facilities became limited in POC testing service because of new NHIA accreditation or renewal was the fact that non-programme POC tests are purchased by CHPS facilities from their service account and claims were made every month to the NHIA for reimbursement of commodities used. Failure to remain accredited subjected CHPS facilities to no reimbursement. A FGD participant interrupted:

[…] *The facility is now limited to only programme commodity tests because the NHIA accreditation has expired and we cannot reclaim any money on tests we may use* […] (FGD Participant 2).

Another FGD participant mentioned:

[…] *We have not gained accreditation from NHIS so no non-programme POC tests can be used here because the facility will not be reimbursed if we use them to use* […] (FGD Participant 9).

#### Stock-outs of POC diagnostic test kits

The analysis revealed that stock-out of POC diagnostic test kits at the depot was a major barrier to POC diagnostic services in low-resourced health facilities. Participants shared their frustrations in the requisition processes; from facility level to district, and region, only to be told that the diagnostic kits were out of stock. A participant reported:

[…] *Anytime we run short of tests, we request through the district health directorate to the regional medical stores (RMS). After going through this procedure, they tell you tests are out of stock. So, we must wait till the regional depot is ready to supply the next available consignment…. This is unbearable!* […] (Interviewee 6).

Another participant indicated:

[…] *We have just a few test kits available. Requisition has been done for almost a month now. Right now we can run out of stock any moment* […] (Interviewee 3).

The study analysis also showed that inadequate funding to procure POC tests also resulted in a stock-out at the CHPS facilities. It was indicated that POC tests such as glucose, Hep B, and Hb are said to be commodities purchased from a service account and the account was persistently empty causing irregular purchases and subsequent stock-outs. A participant reported:

[…] *Both Hep B and glucose strips are out of stock at the moment* […] (Interviewee 3).

Another participant added:

[…] *There are no available funds to purchase POC tests* […] (Interviewee 11).

Upon further investigation, the service account was used for renovations, payment for causal staff, and procurement of commodities, and for that matter, there was always pressure on the service account making POC test purchases irregular and triggering stock-out. A participant exclaimed:

[…] *From the service account, we make payments for casual staff, renovations, and POC test procurement* […] (Interviewee 9).

Another participant added:

[…] *We are using the service account for everything except drugs. There is too much pressure on the account* […] (Interviewee 15).

As to why there was a reimbursement delay by NHIA, participants admitted submitting monthly documentation for claims late because of their busy schedule coupled with inadequate staffing at the CHPS facility.

[…] *As I speak, the facility has not been reimbursed by NHIA for six months. So, there is no fund to make any purchases* […] (Interviewee 4).

Another participant added:

[…] *Our documentation for claims mostly goes to NHIA late because of our staff strength and busy schedules which cause delays in reimbursement* […] (Interviewee 5).

Again, the analysis revealed that the expiring date of POC test kits affected the stock levels at the facilities. Participants reported that commodities near expiring were delivered to CHPS facilities. These commodities expired in a known time and could be used. A FGD participant remarked:

[…] *We sometimes receive commodities near expiration. So, the moment the expiration date is due we can’t use them causing shortages* […] (FGD Participant 9).

Another participant also declared that the RMS gave supplies of non-programme commodities to the CHPS facilities on credit. Failure to make payments on time to the medical stores, commodity shortages occurred because no supplier was willing to bid tender because the medical store was in debt which resulted in in-country shortages. A FGD participant commented:

[…] *We must settle other debtors before the medical stores. We are all government agencies working for the nation* […] (FGD Participant 8).

Another FGD participant added:

[…] *It will be difficult for medical stores to get suppliers to bid tender if they are not able to pay the exorbitant debt owed to suppliers* […] (FGD Participant 3).

Regarding stock-out, it was also indicated that some providers made unreasonable reports and requests on the District Health Information Management System II (DHIMS II*)* which affected the national stock levels of POC tests. A FGD participant mentioned:

[…] *The number of POC test kits used must be well calculated to render an accurate report on the DHIMS II* […] (FGD Participant 3).

Another FGD participant seconded:

[…] *Very true! Some providers make huge requests for commodities without explanation* […] (FGD Participant 4).

Describing the meaning of an unreasonable request, it was emphasized that the CHPS facilities requested a sharp increase in quantity without indicating any upcoming or out-gone supporting activities like the ‘know your status exercise’ of HIV to buttress their huge request rendering a decline of requisition by the depot. A FGD participant reported:

[…] *We decline all inappropriate requests by the CHPS because no CHPS is expected to request more than its average monthly consumption without indicating extra activities such as the “know your status” campaign aside from normal duties. We want to see a reasonable request on the DHIMS II* […] (FGD Participant 4).

As to why the depots declined some requests from CHPS, the analysis revealed that poor reporting of data on DHIMS II affected national quantification leading to national shortage or expiry of test kits country-wide. Another FGD participant explained:

[…] *Programme managers depend on reported data on DHIMS II to quantify programme commodities for the Nation. So, anytime providers give wrongful data (under/over the calculation of stock level) it affects the national quantification. Therefore, anytime there is under calculations or over calculations we see national shortage and expiry of product respectively* […] (FGD Participant 3).

Further on programme commodities, it was highlighted that epidemiological transition attracted the attention of global partners to reduce funding for existing programme-commodity to support outbreaks which affected the supply of programme commodities and caused stock-outs. For instance, a FGD participant remarked:

[…] *Hmm! The COVID-19 pandemic was the biggest experience. The whole nation recorded a stock-out of RDT kits during that time, because donors paid much attention to the pandemic* […] (FGD Participant 5).

Analysis from the discussion on programme commodity also revealed that delays in the port clearance process and documentation resulted in stock-outs. Participants expressed worries about how documentation between the Director of Procurement and Stores and the Procurement Minister delays before it is justified in parliament to gain waiver on the commodities to be cleared from the port before the tests get to the facilities. A FGD participant remarked:

[…] *Anytime we are expecting supplies from oversees, we call the Director of Procurement and Stores but the only answer we get is ‘the commodities have arrived, and we are waiting for a waiver before clearance* […] (FGD Participant 4).

Another FGD participant indicated:

[…] *This waiver and clearance issue delays a lot. Hmm! It can take more than six months and requisitions will be coming to us from* CHPS […] (FGD Participant 3).

#### Supply-related challenges of POC diagnostic test kits

The analysis revealed that supply-related challenges affected POC diagnostic test services in rural settings. Participants indicated that the poor road networks in the region affected the effective supply of commodities, including POC test kits, from the depots to rural facilities. Trucks conveying commodities often hook down requiring toll before journeying to their destinations which affect the supply of POC diagnostic testing services. A participant explained:

[…] *The bad road network in this region affects our distribution; most CHPS are in the hinterland, and roads there are not motorable. Imagine a 15–20 feet truck conveying commodities to these places. The trucks hook down most of the time, especially in the rainy season, and cause a whole lot of delays and extra expenses* […] (Interviewee 1).

Moreover, this research finding indicates that many of the low-resourced facilities have poor road networks, and driving on these roads causes delays making commodities distribution difficult. A FGD participant interjected:

*A long truck is used by the RMS and the poor road network does not permit delivery at our doorsteps* […] (FGD Participant 6).

Another FGD participant exclaimed:

[…] *Yes, commodities are mostly deposited at our prospective district depots for collection* […] (FGD Participant 7).

Another FGD participant added:

[…] *there is also a communication barrier due to unavailable network* […] (FGD Participant 1).

In the analysis, another concern raised indicated that the Last Mile Distribution policy affects the supply of POC test kits to lower-level facilities, resulting in shortages. A FGD participant reported:

[…] *The Last Mile Distribution is causing shortages of POC test kits in this facility* […] (FGD Participant 8).

It was manifested that the Last Mile Distribution policy was implemented using only one long truck to distribute commodities to every health facility in all the districts including CHPS facilities in the hinterland. For this reason, commodities were not supplied regularly. A FGD participant reported:

[…] *We often do not receive test kits as expected. Deliveries take time under the last mile distribution policy* […] (FGD Participant 1).

Another FGD participant added:

[…] *only one truck is used to supply commodities to all facilities in the region* […] (FGD Participant 2).

It was further revealed in the data that providers did not use POC test kits with poor packaging and ripped-off boxes because of a loss of trust in their potency. Due to that some test kits were used and as a result, shortages occurred. A participant complained:

[…] *We experience shortages because we do not use POC tests in tear boxes, wretched wrappings, and spilled buffers solutions because we cannot trust their potency* […] (Interviewee 8).

Again, the analysis showed that unused test kits accounted for shortages of POC test kits because the provider thought that the quantities of buffer solutions provided in the packs were not enough for the number of tests in the pack.

[…] *There are unused test kits because sometimes buffer solutions are not enough for the test kits and* […] (Interviewee 10).

### Identified solutions for POC diagnostic testing implementation barriers

To resolve barriers to POC testing services participants identified and proposed some potential solutions such as adequate funding, effective delivery system, supply of tests to the facilities, and stakeholders’ engagement and advocacy. This is shown in Table 2.

#### Adequate funding

In finding potential solutions to POC diagnostic testing barriers such as supply-related challenges, and stock-out, participants highlighted that adequate funding could help resolve many of the supply-related challenges. Participants emphasized that with adequate funding additional vans which can access roads in remote areas could be purchased to assist in delivering commodities to the facilities. A participant exclaimed:

[…] *Adequate funding is needed to provide additional vans to assist delivery of commodities* […] (Interviewees 9 ).

A FGD participant explained:

[…] *I believe that more vans for delivery will enhance the quick distribution of POC tests to the facilities* […] (FGD Participant 11).

Still, on adequate funding, it was revealed that adequate funding can halt the stock-out rate if NHIS reimbursement delays are not addressed. Participants mentioned that though NHIS may delay if there is adequate funding, health depots can continue to purchase POC diagnostic tests while waiting for payments to maintain stock level. A participant indicated:

[…] *RMS as well as CHPS facilities can still procure commodities when there is adequate funding [*…] (Interviewee 12).

Another participant interjected:

[…] *Delay of reimbursement must not halt POC testing services. We need to have adequate funds* […] (Interviewee 15).

Providing adequate funding was proposed as one of the potential solutions to finance health services which will ensure the effectiveness and smooth running of the health system. Participants believe that with adequate funds POC tests could be bought in large quantities at the depot as well as the facility level to maintain a high stock level and help reduce shortages and stock-out of tests at the facilities. A participant indicated:

“[…] Adequate funds would help mitigate shortages and stock-out […] (Interviewee 16)”.

Another participant added:

[…] Well, with adequate funding facilities and depots can buy in large quantities and minimize the shortage rates […] (Interviewee 9).

The analysis also shows that participants believe that maintaining adequate stock levels will enhance the quick delivery of commodities whenever a requisition is made. A participant indicated:

[…] I *believe improving material resources to maintain adequate stock levels would be excellent in solving POC testing barriers.* […] (Interviewee 16).

Another participant proposed:

[…] *When POC tests are at the depot, our request could be granted on time and the facilities can also maintain their stock levels to avoid shortages* […] (Interviewee 9).

#### Effective delivery system

In the study’s analysis, it was revealed that effective delivery systems have the potential to minimize supply-related barriers to POC test kits, and thus reduce regular stock-outs at the depot and facility levels. Participants reported that the delivery system needs serious improvement for the successful implementation of POC diagnostic services. A FGD participant indicated:

[…] *The supply or delivery services have too many challenges and need to be improved for quality POC services* […] (FGD Participant 6).

Firstly, participants proposed the use of drones to deliver POC tests in remote areas to resolve the barriers related to poor road networks and regular breakdown of lung trucks, especially in hard-to-reach areas. A participant mentioned:

[…] *The cost of constructing and repairing our existing roads might take decades and huge sums by the government. So, if the directorate can switch to using drones for deliveries, POC tests could be delivered safely, and testing could be sustained*. […] (Interviewee 10).

Again, from the study data, restocking the district depots is one way of improving the delivery system for POC test kits to lower-level facilities. Participants indicated that this initiative would ensure the constant supply of POC test kits to low-level facilities in the catchment area of the district, and greatly reduce stock-out issues. A participant indicated:

[…] *I think if the directorate restocks the district depots, access to the tests by the facilities will be quick, and frequent shortages could be reduced* […] (Interviewee 7).

The data further revealed that receiving supplies from considerable proximity would ensure quick access to POC test kits. Another participant suggested:

*[…] The nearness of supplying units to the end user also ensures how quickly commodities can arrive to the end user […]* (Interviewee 14).

Other participants also suggested that an effective monitoring team is needed in every district to improve the delivery system. It was reported that such teams could receive concerns related to supply challenges and resolve them promptly. A participant explained:

[…] *There should be a monitoring team in every district for us to channel our grievances concerning supplies* […] (Interviewee 9).

Another participant added:

[…] *proper monitoring team can also resolve issues relating to supplies such as maintenance of stock levels* […] (Interviewees17).

#### Stakeholder engagement and advocacy

Participants identified stakeholders’ engagement and advocacy in POC testing service implementation as necessary to identify and resolve barriers to POC testing services. Participants encouraged continuous advocacy with major stakeholders such as the global fund to help to increase funding for programme commodities. A FGD participant suggested:

[…] *In my opinion, there is the need to make continuous advocacy to sustain all existing programmes until we achieve the set goals. Let’s continue to talk to global funds to increase its funding* […] (FGD Participant 2).

Another participant proposed from the FGD:

[…] *Stakeholder meeting with the directors of the regional health directorate to explain to them the major reasons for stock-outs, and request for implementation of co-payment whereby the client will be made to do part-payment to supplement their funds to make some purchases before the NHIS reimburses the facilities* […] (FGD Participant 7).

Further to the potential solution to sustain POC diagnostic tests implementation, stakeholders’ engagement was proposed as the best way through which manufacturers would be informed of shortages occurring due to the insufficient buffer solutions in the pack of POC test kits produced. A participant proposed:

[…] *Stakeholder engagement must be used as a channel to inform manufacturers of low buffer solutions* […] (Interviewee 1).

Another participant explained:

[…] *Enough buffer must come with every pack of POC test kits produced to avoid waste and limit shortages.* […] (Interviewee 3).

Moreover, the analysis shows that accreditation challenges leading to POC testing limitations could be resolved when there is active stakeholders’ engagement and advocacy. It was established that major stakeholders such as directors of health service, management of NHIA, and health providers could make conscious decisions to fast-track the NHIA accreditation process to help resolve POC testing limitations at some CHPS facilities. A participant proposed:

[…] *The medical directors and the NHIA management team must meet to make accreditation faster to enhance POC testing scale-up* […] (Interviewee 14).

Another participant exclaimed:

[…] *We cannot offer most of the POC tests when NHIA accreditation has expired because ‘no accreditation no NHIA claims’* […] (Interviewee 16).

#### In-service and refresher training courses

Participants indicated that in-service and refresher training courses would be a potential solution to the POC test implementation barrier. In the analysis, a proposal was raised that in-service training and refresher courses would help improve POC diagnostic testing service because it will equip staff. A participant explained:

[…] *We believe in-service and refresher training courses are very necessary for equipping our staff* […] (Interviewee 5).

Another reason why in-service and refresher course was seen as potential solutions to stock-out was the indication of the periodic reposting of staff and the associated knowledge gap that were created. It was explained that old staff went on transfer or retirement and new staff were replaced. Therefore, to fill the knowledge gap, in-service and refresher training were necessary to avoid wrongful requests and report on the DHIMS II leading to stock-out.

A participant proposed:

[…] *In-service training, as well as a refresher course, must be introduced because periodically, new staff is posted. Old staff may be transferred or retired* […] (Interviewee 7).

A participant from the FGD submitted:

[…] *In-service and refresher training is necessary to refill the knowledge deficit especially, on new brand commodities* […] (FGD Participant 7).

To add to in-service and refresher training courses for especially, the newly posted staff, participants believed it would enable the new staff to learn how to calculate stock level and make proper requests on the DHIMS II. A participant from FGD suggested:

[…] *The officers will learn how to calculate the stock level of this commodity so that it will not run stock-out in the facility* […] (FGD Participant 4).

Another participant from FGD also interjected:

[…] *in-service and refresher training course will empower providers to make correct and accurate requests for POC test on the DHIMS II* […] (FGD Participant 10).

Another participant from the FGD added:

[…] *We can only have accurate reports and requests made on the DHIMS II if regular training is given to staff* […] (FGD Participant 11).

Again, in finding potential solutions to shortages and stock-out, the study found that in-service and refresher would empower staff on how buffer is used on test kits efficiently to yield correct results of tests and avoid shortages. A participant interjected:

[…] *I perceived invalid test strips may be a result of either excess or insufficient use of buffer on strip* […] (Interviewee 6).

Another participant added:

[…] *We may be using more buffer than expected on test strips* […] (Interviewee 16).

## Discussion

This study revealed several barriers including work overloads, stock-out of POC tests, supply-related challenges of POC tests, and limited POC testing service affecting the provision of POC testing services in rural settings. However, potential solutions such as adequate funding, an effective delivery system, stakeholders’ engagement and advocacy, and in-service training and refresher courses were identified. Studies have shown that POC diagnostic testing implementation has contributed immensely to the improvement of health especially, in rural areas [20, 41]. Like Huddy et al’s study, this study found that POC testing provides rural practice with access to diagnostic investigations [20]. This study supported Martin et al’s study on facilitators and barriers to POC testing for sexually transmitted infections in LMICs, and Ansu-Mensah et al’s study on facilitators and barriers to in vitro diagnostics implementation in a resourced-limited setting which reported several barriers to POC testing implementation [16, 17].

The study highlighted some barriers to POC testing implementation not different from previous studies. Work overloads distract the time, energy, and strength needed to carry out various tasks such as POC testing [17, 41, 42]. In a well-structured setting, adequate staff capacity remains a pillar through which employees minimize risk and optimize output as well as improve the talents and skills of staff [43]. Inadequate staffing which potentially resulted in work overloads on staff and the associated difficulties in discharging their duties might have accounted for ineffective testing and wrong diagnosis and treatment. In Ghana, CHPS is considered a pragmatic strategy to achieve the UHC through the provision of essential healthcare basically for women, children, and minor illnesses for the rural people [44–46]. As revealed in the study findings inadequate staff resulted in limited POC testing. This study limitation might have caused frequent referrals of patients particularly, pregnant women needing antenatal screening to high-level facilities [31]. This implies that the CHPS aim of bridging physical and geographical barriers to essential healthcare delivery towards attaining UHC would not be achievable [44–46]. Consequently, the approach of ‘Test, Treat, and Track’ in malaria cases would not be adhered to in facilities where malaria testing was restricted [47, 48]. Another barrier discovered in this study was the stock-out of POC test kits. Providers expressed disappointment in how requisition passed through their prospective district depots to the regional depot before suppliers were received causing regular stock-outs. Stock-outs, as reported in the study, might have resulted in frequent referrals and subsequent patients’ inability to access the POC testing from high-level facilities due to poor geographical access as reported in Ansu-Mensah et al’s study [49]. Moreover, supply-related challenges of POC tests were reported as barriers at both depots and CHPS facilities. A previous study from Ghana that assessed the effectiveness of POC tests for malaria and anaemia reported that a logical analysis of the supply of POC tests could detect supply chain issues [50]. This implies that shortages due to poor supply of tests (poor packaging and ripped boxes of test kits) might have affected effective supply chain management in POC testing implementation in rural areas. Supply-related challenges might have also resulted in shortages or stock-outs due to expiry. For instance, as mentioned in the study findings, many of the facilities in the hinterland were without a communication network and the ineffective Last Mile Distribution policy resulted providers in picking deliveries from their prospective districts. Meanwhile, the Last Mile Distribution aimed to convey medical commodities to the end user [51]. Therefore, providers may not have been informed early enough to pick the commodities when they were deposited in district depots contributing to the expiration of POC tests and consequently, shortages or stock-outs. Some providers may also lack knowledge of how the buffer is used on test kits. This implies that the quantity of buffer on test kits might have been squeezed more than prescribed on test kits resulting in shortages. Again, incorrect use of buffer might have also influenced the potency of test results (positive, negative, or invalid).

The study identified adequate funding as one of the potential solutions to barriers to POC testing implementation. Though supply-related challenges and stock-out were separately reported barriers to POC testing services, adequate funding was mentioned as a potential solution to address them. For instance, a poor supply of POC tests, and stock-out resulting from Last Mile Distribution could be resolved when there are enough funds to purchase additional vans or drones to deliver commodities to the hard-to-reach areas. This would avoid any supply-related and stock-out challenges without going through the hassle of poor road networks. This finding represents a true reflection on Martin et al’s study which investigated facilitators and barriers to POC testing for sexually transmitted infections in low-and middle-income countries where funding and supply chain management were said to be intimately linked [16]. Again, the study identified an effective delivery system as another potential solution to mitigate barriers to POC diagnostic testing services such as supply-related challenges and stock-out. A conscientious effort to build an effective delivery system in the study area requires effective monitoring which would enable providers to channel their grievances to decision-makers to help build a sustainable POC testing service. Participants also felt that restocking the district depots would ensure an effective delivery system and resolve challenges related to the supply of POC tests and stockout at the CHPS facilities. Moreover, the study identified stakeholders’ engagement and advocacy (soliciting ideas and knowledge from key stakeholders) as one potential solution to resolve barriers such as work overload, and limited POC testing service. Just like Shephard et al. discovered in their study, no sustainable POC testing service could be implemented without stakeholders’ engagement [52]. Through stakeholders’ engagement and advocacy, users of POC tests could channel all complaints causing work overload, and limited POC testing services such as accreditation, understaffing, accommodation, etc. to the appropriate stakeholders for possible interventions to resolve challenges to improve and sustain POC testing service. Similarly, stakeholders’ engagement and advocacy were considered a potential solution to resolve delays in port clearance to enhance the quick distribution of POC tests across the country. Lastly, participants suggested that due to periodical transfers and retirements, in-service and refresher training courses would help to update and introduce new staff to existing commodities and their correct usage and reading of test results. Empowering staff through in-service and refresher training courses was considered to bridge the knowledge deficit due to transfers and retirements.

This study provides a piece of pragmatic information on the barriers and potential solutions to POC diagnostic testing in CHPS facilities, to help strengthen, improve, and sustain POC testing implementation in low-resourced settings in the future. To the best of our knowledge, this research is the first to identify barriers and potential solutions to the existing WHO EDL for use in community and healthcare facilities without laboratories that could support policymakers of POC diagnostic testing services in the Bono Region of Ghana. Moreover, the study will serve as a guide for future researchers to investigate POC testing services in rural areas. Nevertheless, the study has some limitations. The study used a single qualitative approach which limits the transferability of the results to future settings. We could have improved flexibility when multiple approaches were used. Again, the study was limited to healthcare providers and depot managers of POC diagnostic tests. Other stakeholders such as patients, opinion leaders, and decision-makers of POC tests may have experience in POC testing challenges and better solutions to mitigate these challenges. Lastly, the study considered facilities in low-resource settings (CHPS). This implies that some higher-level facilities may have a similar experience with POC testing implementations. Therefore, assessing POC testing barriers and potential solutions from other stakeholders such as patients, providers, and decision-makers in higher-level healthcare facilities would have given us a broader view of the study. Notwithstanding these limitations, authors were able to obtain exclusive insight concerning barriers and potential solutions to POC test implementation from healthcare providers, and managers of POC tests in the Bono Region.

## Conclusion

This study has presented barriers to POC testing services implementation and the need to improve and sustain POC testing services at CHPS facilities in the Bono Region. Work overloads, stock-out of POC tests, supply-related challenges of POC tests, and limited POC testing service are the major barriers to successful POC testing services implementation in these facilities in the region. These barriers would therefore, require key stakeholders such as the Ministry of Health, Ghana Health Service, donors, and non-governmental organisations to consider the identified solutions: adequate funding, effective delivery system, stakeholders’ engagement and advocacy, and in-service and refresher training courses found in the study to solve all barriers to build sustainable POC testing services in the Bono Region of Ghana.

### Electronic supplementary material

Below is the link to the electronic supplementary material.

Supplementary Material 1

## Data Availability

The University of KwaZulu-Natal remains the rightful owner of this study’s data which can be made available to all interested persons upon satisfactory acquisition criteria are met through Prof. Themba G. Ginindza the supervisor, or Dr. Desmond Kuupiel the co-supervisor, School of Nursing, and Public Health on the following contact: Ginindza@ukzn.ac.za, and desmondkuupiel98@hotmail.com/Desmondk@dut.ac.za. Alternatively, data for the study could be accessible upon request from the Biomedical Research Ethics Committee BREC), the University of KwaZulu-Natal through the Chairperson Biomedical Research Ethics Administration Office, Westville Campus, Govan Mbeki Building, University of KwaZulu-Natal, P/Bag X54001, Durban 4000 KwaZulu-Natal, South Africa or Tel.: +27312604769 or Email: BREC@ukzn.ac.za. Or. The Chairperson, Ghana Health Service Ethics Review Committee, Research & Development Division, Ghana Health Service, Post Office Box MB 190, Accra. Digital Address: GA-050-3303 or Mob: +233 503 539 896 or Tel.: +233 302 681 109 or Fax: +233 302 685 424 or Email: ethics.research@ghsmail.org.

## Abbreviations

ASSURED: Affordable Sensitive Specific User friendly Rapid and Robust Equipment free and Deliverable
CHPS: Community Health-based and Planning Services
FGD: Focus Group Discussion
HCPs: Healthcare Professionals
LMICs: Lower-and middle-income countries
NCDs: Non-communicable Diseases
MOH: Ministry of Health
NHIA: National Health Insurance Authority
POC: Point-of-care
RMS: Regional Medical Stores
SDGs: Sustainable Development Goals
SSA: Sub-Sahara African Countries
UHC: Universal Health Coverage
UK: United Kingdom
WHO: World Health Organization

## References

[1] Wang YC, Lee YT, Yang T (2020). Current diagnostic tools for coronaviruses-from laboratory diagnosis to POC diagnosis for COVID-19. Bioeng Transl Med.

[2] Heidt B, Siqueira WF, Eersels K. Point of care diagnostics in resource-limited settings: a review of the present and future of PoC in its most needed environment. Biosensors. 2020;10(10). 10.3390/bios10100133 PMC7598644 32987809

[3] Cooke J, Llor C, Hopstaken R, Dryden M, Butler C. Respiratory tract infections (RTIs) in primary care: narrative review of C reactive protein (CRP) point- ­ testing (POCT) and antibacterial use in patients who present with symptoms of RTI. 2020. 10.1136/bmjresp-2020-000624 PMC7476490 32895246

[4] Quinn AD, Dixon D, Meenan BJ (2016). Barriers to hospital-based clinical adoption of point-of-care testing (POCT): a systematic narrative review. Crit Rev Clin Lab Sci.

[5] Ansu-Mensah M, Kuupiel D, Availability VB. Stock Levels and Usage of In-vitro Diagnostics the in Bono Region, Ghana: A Cross-Sectional Study. 2023. 10.4102/phcfm.v15i1.4114 PMC10623501 37916723

[6] Asamani JA, Ismaila H, Plange A. The cost of health workforce gaps and inequitable distribution in the Ghana Health Service: an analysis towards evidence–based health workforce planning and management. Hum Resou Heal. 2021:1–15. 10.1186/s12960-021-00590-3 PMC8010987 33789670

[7] Ankrah AK, Dako-Gyeke P (2021). Factors influencing the delivery and uptake of early infant diagnosis of HIV services in Greater Accra, Ghana: a qualitative study. PLoS ONE.

[8] Palmer T, Aiyenigba AO, Bates I (2020). Improving the effectiveness of point of care tests for malaria and anaemia: a qualitative study across three Ghanaian antenatal clinics. BMC Health Serv Res.

[9] Kuupiel D, Adu KM, Bawontuo V (2020). Geographical access to point-of-care testing for hypertensive disorders of pregnancy as an integral part of maternal healthcare in Ghana. BMC Pregn Childbi.

[10] Kuupiel D, Adu KM, Bawontuo V. Geographical accessibility to glucose-6-Phosphate Dioxygenase Deficiency Point-of-care testing for Antenatal Care in Ghana. Diagnos MDPI. 2020;10(4). 10.3390/diagnostics10040229 PMC7235997 32316233

[11] Boadu NY, Amuasi J, Ansong D (2016). Challenges with implementing malaria rapid diagnostic tests at primary care facilities in a Ghanaian district: a qualitative study. Malar Journ.

[12] Kweku M, Amu H, Awolu A (2020). Community-Based Health Planning and Services Plus programme in Ghana: a qualitative study with stakeholders in two systems Learning districts on improving the implementation of primary health care. PLoS ONE.

[13] World Health Organisation. Second WHO Model List of Essential In Vitro Diagnostics. 2019 1–52.

[14] Naseri M, Ziora ZM, Simon GP. ASSURED-compliant point‐of‐care diagnostics for the detection of human viral infections. Revie Med Virolo. 2021;32(2).

[15] Smith S, Korvink JG, Mager D (2018). The potential of paper-based diagnostics to meet the ASSURED criteria. Roy Socie Chemi.

[16] Martin K, Wenlock R, Roper T (2022). Facilitators and barriers to point-of-care testing for sexually transmitted infections in low- and middle-income countries: a scoping review. BMC Infect Dis.

[17] Ansu-Mensah M, Kuupiel D, Asiamah EA (2023). Facilitators and barriers to in vitro diagnostics implementation in resource-limited settings: a scoping review. Afric Journ Pri Health Care Fami Medi.

[18] World Health O. First WHO Model List of Essential In Vitro Diagnostics2019. 1–73 p.

[19] Nations U. The 2030 Agenda and the Sustainable Development Goals An opportunity for Latin America and the Caribbean. 2018.

[20] Huddy JR, Ni MZ, Barlow J (2021). Qualitative analysis of stakeholder interviews to identify the barriers and facilitators to the adoption of point-care diagnostic tests in the UK. BMJ open.

[21] Shahid I, Alzahrani AR, Al-Ghamdi SS, Hepatitis C. Diagnosis: Simplified Solutions, Predictive Barriers, and Future Promises. Diagnos MDPI. 2021;11(7). 10.3390/diagnostics11071253 PMC8305142 34359335

[22] Engel N, Ganesh G, Patil M (2015). Barriers to point-of-care testing in India: results from qualitative research across different settings, users and major diseases. PLoS ONE.

[23] Pai NP, Steben M. Commentary point of care technologies for HIV/STBBI: an analysis of contextual factors impeding implementation in Canada. Nat Collab Cent Infect Dis. 2018:1–12.

[24] Pai NP, Vadnais C, Denkinger C. Point-of-care testing for infectious diseases: Diversity, Complexity, and barriers in low- and Middle-Income Countries. PLoS Med. 2012;9(9). 10.1371/journal.pmed.1001306 PMC3433407 22973183

[25] Hampanda KM, Nimz AM, Abuogi LL (2017). Barriers to uptake of early infant HIV testing in Zambia: the role of intimate partner violence and HIV status disclosure within couples. AIDS Res Ther.

[26] Hardy V, Thompson M, Alto W. Exploring the barriers and facilitators to use of point of care tests in family medicine clinics in the United States. BMC Fami Practi. 2016:1–8. 10.1186/s12875-016-0549-1 PMC5093922 27809865

[27] Kuupiel D, Tlou B, Bawontuo V, Mashamba-Thompson TP. Accessibility of Point-of-Care Diagnostic Services for Maternal Health in Lower Healthcare Facilities in Northern Ghana: A Cross-Sectional Survey. Available at SSRN 3204717. 2018.

[28] Van Ginderdeuren E, Bassett J, Hanrahan C (2019). Health system barriers to implementation of TB preventive strategies in South African primary care facilities. PLoS ONE.

[29] Van GY, Onasanya A, Van Engelen J. Improving access to diagnostics for schistosomiasis case management in oyo state, Nigeria: barriers and opportunities. Diagnos MDPI. 2020;10(5). 10.3390/diagnostics10050328 PMC7278006 32443849

[30] Jacobs B, Ir P, Bigdeli M. Addressing access barriers to health services: an analytical framework for selecting appropriate interventions in low-income Asian countries. Heal Poli Plan 2012(May 2011):288–300. 10.1093/heapol/czr038 21565939

[31] Kuupiel D, Manu K, Bawontuo V (2019). Geographical accessibility to District Hospitals / Medical Laboratories for Comprehensive Antenatal Point-of-Care Diagnostic Services in the Upper East. EClinicalMedi.

[32] Kuupiel D, Boikhutso T, Vitalis B (2019). Poor supply chain management and stock-outs of point-of-care diagnostic tests in Upper East Region’s primary healthcare clinics, Ghana. PLoS ONE.

[33] Kuupiel D, Adu KM, Apiribu F. Geographic accessibility to public health facilities providing tuberculosis testing services at point-of-care in the upper east region, Ghana. BMC Publ Heal 2019:1–12. 10.1186/s12889-019-7052-2 PMC6558903 31182068

[34] Chatio ST, Duah E, Ababio LO (2022). Barriers and facilitators to Community Acceptability of integrating point-of-care testing to screen for Sickle Cell Disease in Children in Primary Healthcare settings in Rural Northern Ghana. Blood.

[35] Caleb L, Ward, Marissa Z, Guo, Amukele TK. Availability and prices of WHO Essential Diagnostics in Laboratories in West Africa: a Landscape Survey of Diagnostic Testing in Northern Ghana. The Journ of Appli Labo Med; 2021. 10.1093/jalm/jfaa190 33438734

[36] Sinha A, Abdul-Mumin A, Abubakari A. Field evaluation of specificity and sensitivity of a standard SARS-CoV-2 antigen rapid diagnostic test: a prospective study at a teaching hospital in Northern Ghana. PLOS Global Public Health. 2021;1(12). 10.1371/journal.pgph.0000040 PMC10021174 36962111

[37] Dassah Edward Tieru YA-S, Mayaud P. Factors Associated with Failure to Screen for Syphilis during Antenatal Acare in Ghana: A Case Control Study. BMC Infectious Diseases [Internet]. 2015; 15(1):[125 p.]. https://bmcinfectdis.biomedcentral.com/track/pdf/10.1186/s12879-015-0868-1.pdf. 10.1186/s12879-015-0868-1 PMC4364573 25888254

[38] Osei E, Nkambule SJ, Vezi PN. Systematic review and Meta-analysis of the diagnostic accuracy of Mobile-Linked Point-of-Care Diagnostics in Sub-saharan Africa. Diagn (Basel). 2021;11(6). 10.3390/diagnostics11061081 PMC8231511 34204848

[39] Mashamba-Thompson TP, Jama NA, Sartorius B (2017). Implementation of point-of-Care Diagnostics in Rural Primary Healthcare clinics in South Africa: perspectives of key stakeholders. Diagn (Basel).

[40] Ansu-Mensah M, Bawontuo V, Kuupiel D, Ginindza T. Focus Group Discussion Guide. 2023.

[41] Wexler C, Kamau Y, Muchoki E (2021). Implementing at-birth, point-of-care HIV testing in Kenya: a qualitative study using the Consolidated Framework for implementation research. Implement Sci Commun.

[42] Young N, Achieng F, Desai M (2019). Integrated point-of-care testing (POCT) for HIV, syphilis, malaria and anaemia at antenatal facilities in western Kenya: a qualitative study exploring end-users’ perspectives of appropriateness, acceptability and feasibility. BMC Health Ser Res.

[43] Maluleke K. A scoping review protocol for Supply Chain Management Systems for Point of Care Diagnostics Services: Optimising COVID-19 Testing Capacity in Resource-limited settings. Resear Squa 2020:1–11. 10.3390/diagnostics11122299 PMC8700402 34943536

[44] Assan A, Takian A, Aikins M, Akbarisari A (2019). Challenges to achieving universal health coverage through community-based health planning and services delivery approach: a qualitative study in Ghana. BMJ Open.

[45] Johnson FA, Frempong-Ainguah F, Matthews Z, Harfoot AJ, Nyarko P, Baschieri A (2015). Evaluating the impact of the community-based health planning and services initiative on uptake of skilled birth care in Ghana. PLoS ONE.

[46] Nyonator FK, Awoonor-Williams JK, Phillips JF, Jones TC, Miller RA (2005). The Ghana community-based health planning and services initiative for scaling up service delivery innovation. Health Policy Plan.

[47] Amartey AO, Buabeng KO, Mohammed A. Quality of Malaria and HIV Rapid Diagnostic Test kits (RDTs) in Health Facilities and Medicines outlets in the Greater Accra Region of Ghana. Interna Journ of Multidisc Res and Analys; 2021.

[48] Oteng G, Kenu E, Bandoh DA (2020). Compliance with the WHO strategy of test, treat and track for malaria control at Bosomtwi District in Ghana. Ghana Med J.

[49] Ansu-Mensah M, Kuupiel D. TG. G. Geographical Access to Point-of-Care Diagnostic Tests for Diabetes, Anaemia, Hepatitis B, and Human Immunodeficiency Virus in the Bono Region, Ghana. 2023. 10.1186/s12913-024-11830-2 PMC11520372 39472915

[50] Palmer T, Aiyenigba AO, Bates I (2020). Improving the effectiveness of point of care tests for malaria and anaemia: a qualitative study across three Ghanaian antenatal clinics. BMC Heal Serv Res.

[51] Atinga O, Pisa N, Walters J. A comparative study of the public and private medical commodity supply chains in Ghana, the case of lastmile delivery in the Upper East Region. 2021.

[52] Shephard M, Shephard A, Matthews S (2020). The benefits and challenges of point-of-care testing in rural and remote primary care settings in Australia. Arch Pathol Lab Med.

