# Supplementary material for: Sustainable solutions to barriers of point-of-care diagnostic testing services in health facilities without laboratories in the Bono Region, Ghana: a qualitative study
Source: BMC Prim Care. 2024 May 22;25:179. doi: 10.1186/s12875-024-02406-4 (PMC11110428; doi:10.1186/s12875-024-02406-4)
Supplement: Supplementary file 1 — Supplementary Material 1 [file 12875_2024_2406_MOESM1_ESM.docx]

***University of KwaZulu-Natal, Durban, School of Nursing and Public Health, Discipline of Public Health Medicine***

***Title:* Evaluating the Accessibility and Barriers and Potential Solutions to Implementation/Sustainability of Point-of-Care Diagnostic Testing Services at the Primary Healthcare Level**

## Focus Group Discussion Guide for:

Facility in-charges/District/Regional Depot Managers of POC Test Focus Group Discussion

1. Barriers to POC diagnostic testing.
2. In your opinion, what difficulties do you face when providing POC testing in your facility?
3. How do these difficulties manifest while providing POC services?
4. Why do these difficulties exist?
5. Sustainable solutions
6. In your opinion, what are possible solutions to these difficulties?
7. How can we implement your suggestions?
8. Why do you think these solutions are sustainable?
